# Clinical and sensory evaluation of an instant carbohydrate beverage as a clear liquid in the preoperative period: randomized controlled trial

**DOI:** 10.1016/j.bjane.2026.844785

**Published:** 2026-06-25

**Authors:** Joice Carla Dorneles Zanin, Rosicler Colet, Marcieli Peruzzolo, Geciane Toniazzo Backes, Jamile Zeni, André Keng Wei Hsu

**Affiliations:** a Universidade Regional Integrada do Alto Uruguai e Missões, URI Erechim, Escola de Medicina, Erechim, RS, Brazil; b Universidade Regional Integrada do Alto Uruguai e Missões, URI Erechim, Programa de Pós-Graduação em Engenharia de Alimentos, Erechim, RS, Brazil; c Clinical Expert Solutions, LTDA, Erechim, RS, Brazil

**Keywords:** Anxiety, Carbohydrates, Coffee, Enhanced recovery after surgery, Fasting, Preoperative care

## Abstract

**Objective:**

Preoperative carbohydrate-containing clear liquids are recommended to reduce discomfort associated with prolonged fasting, yet their implementation remains limited in many Brazilian hospitals. This study evaluated the sensory acceptance and clinical effects of an instant coffee-flavored carbohydrate beverage administered during the preoperative period.

**Methods:**

A randomized controlled trial was conducted between March 2022 and December 2024 at a tertiary hospital in Brazil. Adult patients scheduled for elective surgery under general anesthesia were allocated to a test group (200 mL carbohydrate beverage up to two hours before anesthesia induction) or a control group (conventional fasting of approximately 8 hours). Clinical assessments were performed approximately two hours before anesthesia induction. The primary outcome was satiety, assessed using a 0‒10 Numerical Rating Scale (NRS). Secondary outcomes included thirst and anxiety. In the test group, palatability, satisfaction, and perceived anxiety control were also evaluated.

**Results:**

The beverage demonstrated good sensory acceptance and was well tolerated. Compared with conventional fasting, participants who received the beverage reported significantly higher satiety scores and lower levels of thirst and anxiety. Among participants with higher baseline anxiety (≥ 7), exploratory descriptive findings suggested favorable perceived anxiety control following beverage intake. No adverse events were observed during the study period; however, the study was not powered to detect rare adverse events.

**Conclusion:**

Administration of the beverage up to two hours before anesthesia was associated with reduced preoperative discomfort and high patient acceptance. Larger studies are needed to further evaluate clinical outcomes and safety.

## Introduction

Preoperative fasting was institutionalized in the 1940s by Curtis Lester Mendelson with the aim of preventing pulmonary complications, vomiting, regurgitation, and aspiration of gastric contents during surgery.^1^ For decades, prolonged fasting was considered a standard safety measure. However, beginning in the 1990s, accumulating scientific evidence demonstrated that extended fasting could be detrimental to patients. Consequently, evidence-based perioperative care models such as Enhanced Recovery After Surgery (ERAS) were developed, recommending abbreviation of preoperative fasting as part of a broader multimodal strategy to attenuate surgical stress and accelerate postoperative recovery.^2^, ^3^, ^4^

In parallel, research has shown that prolonged fasting is associated with significant patient discomfort, including hunger, thirst, and anxiety. Moreover, it contributes to metabolic disturbances such as insulin resistance and impaired glycemic control. Excessive caloric restriction may also predispose vulnerable individuals to nutritional deterioration, thereby negatively affecting clinical outcomes.^5^^,^^6^ Thus, the traditional “nil per os after midnight” practice has progressively been reconsidered.

Currently, guidelines from the American Society of Anesthesiologists (ASA) permit the intake of clear liquids up to two hours before elective anesthesia.^7^ Importantly, adequate preoperative nutritional status has been associated with reductions in morbidity, mortality, and healthcare costs, while targeted nutritional interventions contribute to improved recovery.^8^ Furthermore, carbohydrate administration in the preoperative period has been shown to reduce hunger and thirst without increasing the risk of bronchial aspiration.^9^^,^^10^ From a metabolic perspective, prolonged fasting leads to glycogen depletion, decreased insulin secretion, and increased glucagon levels, thereby exacerbating insulin resistance and amplifying the metabolic stress response to surgery.^11^

Taken together, these physiological and subjective effects, including hunger, thirst, discomfort, nausea, and vomiting, may compromise patient well-being and tolerance to fasting. Therefore, ERAS protocols recommend the use of carbohydrate-rich beverages before various surgical procedures to mitigate the adverse consequences of extended fasting.^7^^,^^9^ In this context, the present study aimed to develop a fat-free, carbohydrate-enriched instant beverage based on decaffeinated coffee and to clinically evaluate its effects on hunger, thirst, and anxiety during the preoperative period.

## Methods

This study was conducted in two distinct phases: 1) Product development and characterization (preclinical phase) and 2) Clinical validation through a randomized controlled trial (clinical phase). The study followed the CONSORT guidelines for reporting randomized clinical trials.

### Preclinical phase – instant coffee development and characterization

The instant coffee, free of solids, for the perioperative period was prepared with different concentrations of coffee, maltodextrin, and glucose, resulting in four product Formulations (F1, F2, F3, and F4), all providing 300 kcal per 200 mL serving. Detailed information regarding formulation composition and product development is available in the patent registered with the National Institute of Industrial Property (INPI), n° BR102022022016-6 (https://busca.inpi.gov.br/pePI/servlet/PatenteServletController?Action=detail&CodPedido=1682210&SearchParameter=BR%2010%202022%20022016%206%20%20%20%20%20%20&Resumo=&Titulo=).

For the characterization of the formulations, microbiological analyses for *Salmonella* spp. and *Escherichia coli* were initially performed in accordance with the requirements established by Brazil’s Normative Instruction n° 161, dated July 1, 2022, followed by sensory evaluation.^12^

The sensory evaluation aimed to identify the most acceptable formulation based on taste and overall preference and was approved by the Ethics Committee (CAAE 67767223.9.0000.5351). Sample size was defined according to methodological recommendations for preference ranking tests analyzed using the Friedman test (α = 5%; power = 80%), resulting in the inclusion of 45-untrained volunteers of both sexes who were regular consumers of the product category. Panelists received approximately 30 mL of each formulation at 60°C, served in balanced order in containers coded with random three-digit numbers. Mineral water at room temperature was provided for palate cleansing between samples. Sensory preference was evaluated using a ranking test.^13^ Panelists were instructed to order the four Formulations (F1‒F4) according to overall preference, assigning rank 1 to the most preferred sample and rank 4 to the least preferred. Ranking data were analyzed using the Friedman test to compare differences in mean ranks among formulations. Lower mean rank values were interpreted as indicating higher preference.

The formulation with the highest sensory acceptance was physicochemically characterized for pH, acidity, moisture, protein, lipids, fibers, ash, carbohydrates by difference, energy value, water activity, and instrumental color (L*, a*, and b*), and was subjected to a clinical trial to validate its efficacy. The pH measurement was performed using a pH meter (Digimed, model DM-22, São Paulo, Brazil) calibrated with buffers at 4.0, 7.0, and 10.0. Acidity was determined volumetrically using 0.1 M NaOH solution, and results were expressed in g/100 g on a dry basis. Moisture was determined in a forced-air oven (Fanem 320-SE, São Paulo, Brazil) using 3.0 g of sample at 105°C until constant weight. Protein content was measured by the Kjeldahl method using a digestion-distillation system (VELP-UDK 126A). Lipids were determined by Soxhlet extraction (Nova Ética®, model NT340) using petroleum ether (Química Moderna® 30–60°C). Ash content was determined by combustion of organic matter in a muffle furnace (Lavoisier, model 400C) at 550°C for 6 hours. Water activity was measured using a water activity analyzer (Novasina, Labtouch), calibrated with deionized water and NaCl solution with 0.819 a_w_ until stabilization, followed by measurement of the sample’s a_w_/T (°C). All methodologies followed the Instituto Adolfo Lutz.^14^ Total dietary fiber was determined according to method 985.29.^15^

Color was determined using a colorimeter (Minolta Chroma Meter, model CR-400) in the CIELAB color system, including the three components: L* (lightness or brightness), which ranges from 0 (black) to 100 (white), and the chromaticity coordinates a* and b*, which range from -a* (green) to +a* (red) and from -b* (blue) to +b* (yellow), respectively.

### Clinical Phase ‒ Clinical validation through a randomized controlled trial

This randomized controlled trial was registered in the Brazilian Registry of Clinical Trials (ReBEC; RBR-5qczxhb; https://ensaiosclinicos.gov.br/rg/RBR-5qczxhb) on May 13, 2025. The study was approved by the Human Research Ethics Committee (CAAE 67767223.9.0000.5351) and conducted in accordance with the Declaration of Helsinki. All participants provided written informed consent prior to enrollment.

This phase included real adult patients aged 18‒65 years who were scheduled for elective surgical procedures at the study hospital (Unimed Erechim Hospital, located in the north of Rio Grande do Sul). The trial was conducted between March 2022 and December 2024. Participants were recruited during routine preoperative evaluation visits. All eligible patients received detailed verbal and written information about the study objectives, procedures, and potential risks before signing the informed consent form. Surgeries were performed according to the hospital’s standard schedule (morning or afternoon), and the timing of beverage administration was individually adjusted to ensure ingestion up to two hours before anesthesia induction.

Sample size was calculated based on the primary outcome, defined as the satiety score measured on a 0‒10 numerical scale. Assuming a minimum clinically relevant difference of 1-point between groups, a standard deviation of 1.0 (based on pilot data), a two-sided alpha level of 5%, and 80% statistical power, at least 17 participants per group were required. To increase robustness and account for potential losses, 30 participants were included in each group, totaling 60 patients.

**Figure 1:**
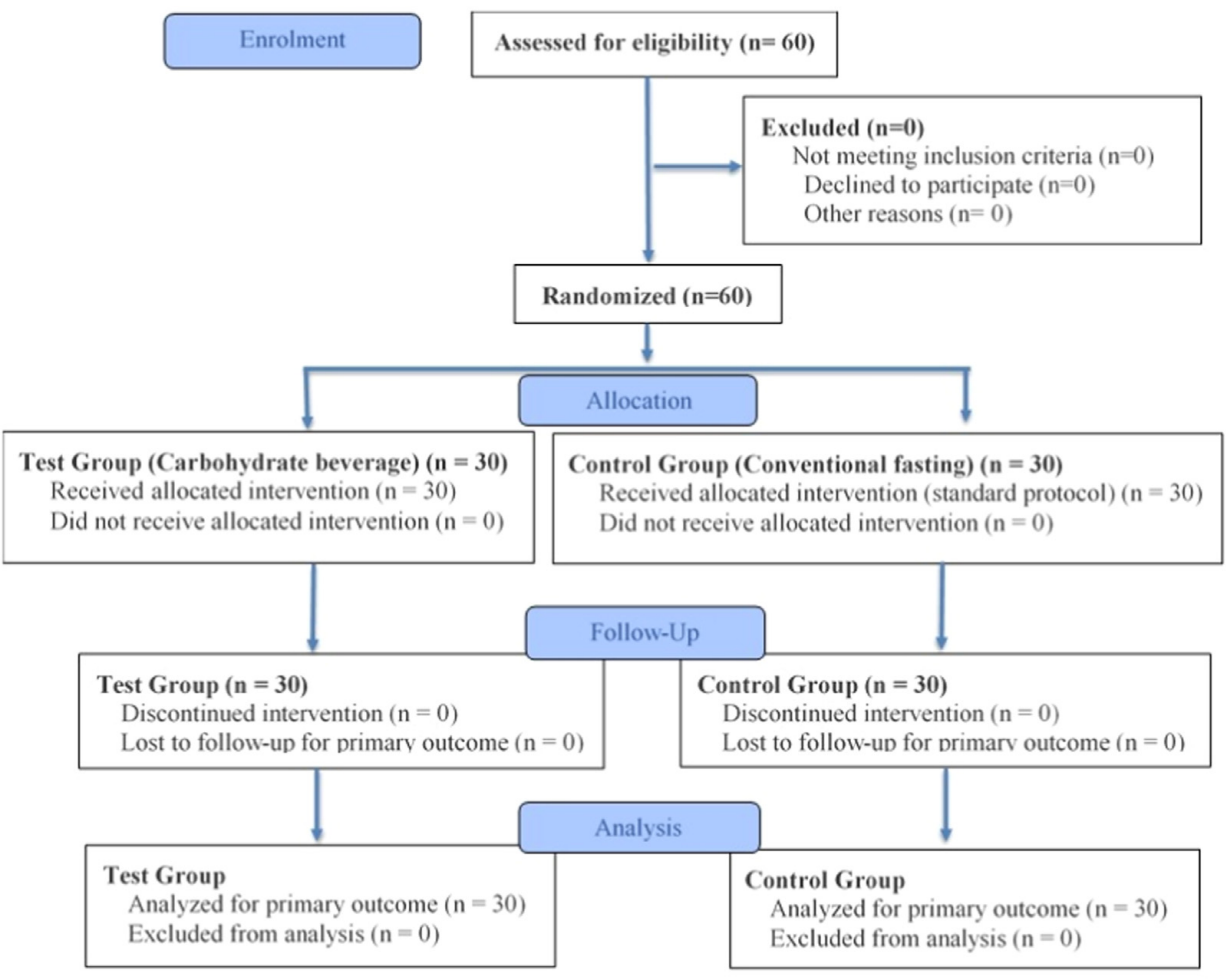
CONSORT flow diagram of the clinical trial on preoperative instant coffee administration, comparing test and control groups.

Participants were randomly allocated in a 1:1 ratio to either the test group (n = 30), which received the instant coffee-based carbohydrate beverage up to two hours before anesthesia induction, or the control group (n = 30), which followed the standard institutional preoperative fasting protocol. The allocation process is presented in the CONSORT flow diagram (Fig. 1). Due to the nature of the intervention, participant blinding was not feasible.

Eligibility criteria were established a priori. Inclusion criteria comprised adults aged 18‒65 years who were candidates for elective surgery and able to understand and complete the study assessments. Exclusion criteria included diabetes mellitus (type 1 or type 2), uncontrolled metabolic or endocrine disorders, gastroparesis, severe gastroesophageal reflux disease, pregnancy, insulin use, use of medications affecting gastric emptying, or increased risk of aspiration as determined by the anesthesiology team. Patients with diabetes were not included in the study.

All surgical procedures were performed under general anesthesia, according to institutional clinical protocols and at the discretion of the attending anesthesiologist. No regional or combined anesthesia techniques were used in this study.

The 200 mL carbohydrate beverage was prepared according to patent BR102022022016-6, following a standardized preparation protocol to ensure consistent dilution, volume, and temperature. The solution was prepared by trained research personnel and administered under supervision in the hospital’s preoperative area. Participants allocated to the test group consumed the beverage up to two hours before anesthesia induction, in accordance with ERAS recommendations. The control group followed the institutional standard fasting protocol, which required a minimum of 8 hours of fasting from the last oral intake (including liquids) until anesthesia induction.

Clinical evaluation was performed at a single preoperative time point, approximately two hours before anesthesia induction. Participants were assessed for sensation of hunger, thirst, anxiety, and related perceptions using structured instruments.

Anxiety was assessed using a 0‒10 Numerical Rating Scale (NRS), where 0 represented “no anxiety” and 10 represented “maximum anxiety”. The NRS is widely used in clinical research and practice because of its simplicity, ease of application, and reproducibility. Thirst was evaluated as a binary outcome (present/absent) based on participants’ self-reports at the time of assessment.

Additional evaluations were performed in the test group following beverage consumption. Palatability was rated using a three-category scale (“very good”, “good”, or “bad”), while overall satisfaction with the intervention was recorded as a yes/no response. Participants also rated their perceived ability to control anxiety after beverage intake on a 0‒10 NRS, with higher scores indicating greater perceived anxiety control.

In the test group, additional assessments were conducted after beverage consumption and prior to anesthesia induction. Approximately 20‒30 minutes after intake, and still within the preoperative holding area, participants evaluated palatability, satisfaction with the intervention, and perceived anxiety control using structured instruments. All assessments were completed before transfer to the operating room.

The numerical and categorical scales used in this study were simple structured instruments developed by the authors for clinical applicability in the preoperative setting and were not previously validated as formal psychometric tools. However, the 0‒10 NRS format is widely accepted and commonly used in clinical research for subjective symptom measurement.

All questionnaires were administered and recorded by trained members of the research team following standardized procedures. Outcomes related to discomfort, hunger, thirst, and anxiety were self-reported by the patients. Sociodemographic and clinical data were collected from medical records and complementary patient interviews.

Potential risks associated with preoperative carbohydrate ingestion, such as delayed gastric emptying or aspiration, were minimized through strict exclusion criteria and adherence to the two-hour fasting interval. All surgeries were performed as scheduled, with no cancellations related to the intervention, and no adverse events associated with beverage ingestion were observed.

### Statistical analysis

Data were analyzed using descriptive and inferential statistics as appropriate. The normality of continuous variables was assessed using the Shapiro-Wilk test. As normal distribution assumptions were not satisfied, non-parametric methods were applied for the analysis of clinical outcomes.

Physicochemical analyses (n = 3) were evaluated using one-way analysis of variance (ANOVA), followed by Tukey’s post hoc test, adopting a 5% significance level. Statistical analyses were performed using Statistica version 5.0 (Statsoft Inc., USA).

For the sensory evaluation stage, differences in preference rankings among formulations were analyzed using the Friedman test. In the clinical phase, between-group comparisons for the primary outcome (satiety score) and other continuous variables were performed using the Mann-Whitney *U*-test. Associations between categorical variables were assessed using Fisher’s exact test.

An exploratory descriptive analysis was conducted among participants in the test group with baseline anxiety scores ≥ 7, defined as moderate-to-high anxiety on the 0‒10 numerical rating scale. Perceived anxiety control after beverage intake in this subgroup was summarized using descriptive statistics only. Owing to the limited number of participants, no inferential statistical testing was performed for this analysis, and findings should be interpreted as exploratory.

## Results

### Product characterization

Microbiological analyses did not detect the presence of *Salmonella* sp. or *Escherichia coli*, confirming that the product meets food safety standards and can be consumed without health risks.

In the sensory analysis stage, 45 panelists participated. Demographic characterization revealed a predominance of females (75%), a higher concentration in the 18‒20 year age range (47%), and a majority educational level corresponding to higher education (56%).

The preference ranking test showed limited variation among formulations with different coffee concentrations. Sample F3 presented the lowest mean rank (2.14), indicating the highest preference among panelists, followed by F4 (2.30), F1 (2.50), and F2 (3.07). Based on these findings, formulation F3 was selected for the clinical phase, considering both sensory performance and technical criteria, including ingredient balance, lower perceived bitterness, and compliance with the patented formulation (BR102022022016-6).

The physicochemical analysis of formulation F3 showed a pH of 5.12 (±0.005), acidity of 9.61% (±0.92), water activity of 0.38 (±0.02), and moisture content of 0.60% (±0.004), indicating stability and low availability of free water. In the instrumental color analysis, the Lightness (L) value was 60.77, classifying it as visually clear with no perceptible residues, making it compatible with the recommendations for clear liquids during preoperative fasting.

The nutritional composition (Table 1), prepared in accordance with RDC n°359 and IN n° 75 (December 23, 2003),^16^ indicates that each 78.53 g serving provides 76.6 g of carbohydrates, corresponding to 25.52% of the recommended daily value. According to the formulation, these carbohydrates are composed of 64.53 g of glucose and 12.07 g of maltodextrin per serving. The formulation contains no fat and is designed to provide rapid energy availability.

**Table 1:** Nutritional information of the instant coffee-flavored product.

Component	Per 100 g	Per serving (78.53 g)	%DV^a^
Energy (kcal)	389.94	306.22	15.31
Total carbohydrates (g)	97.54	76.60	25.52
Glucose (g)^b^	83.44	64.53	–
Maltodextrin (g)^b^	14.10	12.07	–
Protein (g)	0	0	0
Total fat (g)	0	0	0
Saturated fat (g)	0	0	0
Trans fat (g)	0	0	0
Dietary fiber (g)	0	0	0

a % Daily Values based on a 2,000 kcal (8,400 kJ) diet.

b Carbohydrate distribution according to formulation described in patent BR102022022016-6.

Thus, considering the sensory acceptability of formulation F3, its physicochemical suitability, and nutritional value, it is configured as a viable alternative for use in clinical interventions during the preoperative period.

### Clinical trial

In the clinical phase, the test group consisted of 30 participants (27 females and 3 males; mean age 37.5 ± 11.15 years). The most frequently reported comorbidities were systemic arterial hypertension and controlled hypothyroidism, with no significant differences between groups. In the test group, 8 patients (26.7%) had at least one comorbidity, whereas in the control group, 9 patients (30%) reported comorbid conditions, predominantly hypertension, as shown in Table 2. The use of continuous medications reflected these underlying conditions and did not differ significantly between groups.

**Table 2:** Baseline demographic and clinical characteristics of participants.

Variable	Control (n = 30)	Test (n = 30)	p-value
Age (years), mean ± SD	39.96 ± 11.38	37.50 ± 11.15	0.39^a^
Female sex, n (%)	16 (53%)	27 (90%)	0.002^b^
Any comorbidity, n (%)	9 (30%)	8 (26.7%)	0.77^b^
Continuous medication use, n (%)	12 (40%)	9 (30%)	0.41^b^

a Mann-Whitney test.

b Fisher’s exact test.

Based on the data in (Table 3), most participants in the test group reported complete satiety after consuming the product, and 96% experienced relief from thirst. Palatability was well-rated: 60% described it as “very good” and 40% as “good,” with no cases of rejection. Average scores for “anxiety level” and “anxiety control” were 6.33 and 8.23, respectively. Furthermore, all participants expressed satisfaction with the experience, indicating full acceptance of the intervention.

**Table 3:** Preoperative self-reported outcomes.

Variable	Control (n = 30)	Test (n = 30)	p-value
Satiety (0‒10), mean ± SD	6.43 ± 1.07	9.03 ± 0.88	< 0.001^a^
Thirst present, n (%)	29 (96.7%)	1 (3.3%)	< 0.001^b^
Anxiety level (0‒10), mean ± SD	9.00 ± 0.87	6.33 ± 1.70	< 0.001^a^
Anxiety control (0‒10), mean ± SD	–	8.23 ± 0.97	–

a Mann-Whitney test.

b Fisher’s exact test.

**Figure 2:**
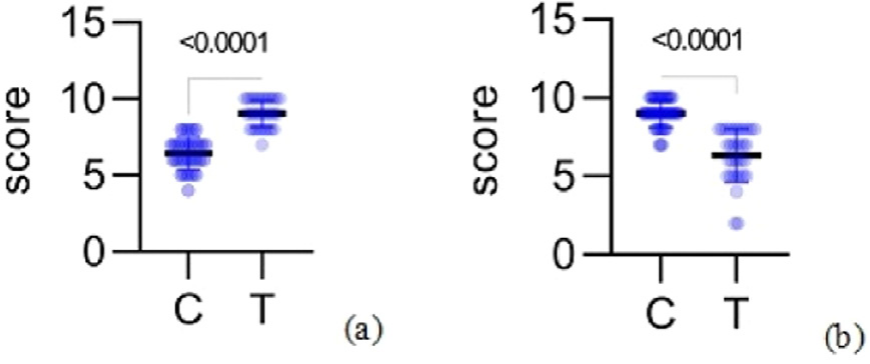
Comparison of satiety between the test group and the control group (a). Comparison of anxiety levels between the test group and the control group (b).

In contrast, the control group showed lower satiety and greater preoperative discomfort, mainly due to hunger and anxiety. When comparing groups, the test group demonstrated significantly higher satiety scores and lower anxiety levels compared to the control group (p < 0.001 for both comparisons), as detailed in Table 3 and Figure 2a. Similarly, mean anxiety levels were significantly lower in the test group compared to the group that remained on conventional fasting (p < 0.001), demonstrating the positive effect of the nutritional intervention on reducing preoperative anxiety (Fig. 2b).

An additional exploratory analysis was performed among participants in the test group who presented baseline anxiety scores ≥ 7. In this subgroup, the mean perceived anxiety control score after beverage intake was 8.38 on a 0‒10 scale, indicating a high level of subjective anxiety control. These findings suggest that even individuals with higher initial anxiety levels reported substantial perceived benefit following the intervention. However, due to the exploratory nature of this analysis and the limited number of participants in this subgroup, these results should be interpreted with caution.

## Discussion

Several national and international scientific societies, such as the American Society of Anesthesiologists (ASA) and the Brazilian Society of Parenteral and Enteral Nutrition (SBNPE), recommend the adoption of multimodal ERAS protocols, including the abbreviation of preoperative fasting time.^17^^,^^18^ However, its practical implementation remains limited in many centers, mainly due to the lack of viable and accessible strategies that combine safety, cost-effectiveness, and patient acceptance.

The ERAS protocol is an evidence-based perioperative care approach aimed at improving the management of patients undergoing surgery. Initially applied in colorectal procedures, its use has expanded to various surgical specialties, showing positive outcomes. The adoption of ERAS has proven effective in reducing hospital length of stay, increasing the safety and efficiency of surgical procedures, and contributing to lower hospital costs.^3^^,^^19^

In this context, the development of a coffee-flavored, instant-reconstitution product that is safe and palatable represents an alternative to conventional preoperative fasting. Clinical analysis showed that consumption of the product up to two hours before elective surgery was associated with improved satiety and thirst control, symptoms frequently reported by patients subjected to prolonged fasting.^9^^,^^20^^,^^21^ No cases of pulmonary aspiration or other adverse events were observed among participants who consumed the product during the study period. However, these findings should be interpreted descriptively and not as definitive evidence of safety.

Furthermore, a significant reduction in anxiety levels was observed in the test group, reinforcing evidence that carbohydrate administration in the preoperative period can alleviate emotional discomfort and contribute to a more positive perioperative experience.^22^, ^23^, ^24^ An exploratory analysis focusing on participants with higher baseline anxiety levels (≥ 7) demonstrated high perceived anxiety control after beverage intake. Although no inferential statistical analysis was performed due to the limited subgroup size, the descriptive findings suggest that the intervention may offer subjective emotional benefits even in more anxious individuals. Future studies with larger samples and repeated anxiety measurements are needed to better characterize this potential association.

An additional exploratory analysis suggested that participants with higher preoperative anxiety levels tended to report greater perceived anxiety control after consumption of the beverage. A positive trend was observed between baseline anxiety intensity and perceived anxiety control scores, indicating that more anxious individuals may experience greater subjective benefit from the intervention. Although this observation does not allow causal inference and was not subjected to formal statistical testing due to the limited sample size, it supports the hypothesis that abbreviated fasting combined with preoperative carbohydrate administration may contribute not only to the reduction of physical discomfort but also to an improved emotional experience during the preoperative period. These findings are consistent with previous studies reporting that carbohydrate-containing beverages administered before surgery are associated with improvements in subjective well-being, comfort, and patient satisfaction. Future studies using larger samples and validated anxiety assessment instruments are warranted to better characterize the magnitude and clinical relevance of this potential association.

The product's palatability was satisfactory and well accepted by all participants, an essential aspect for adherence to the proposed nutritional strategy.^25^^,^^26^ In this regard, it is also noteworthy that the majority of volunteers were female, which may be related to a greater willingness among women to accept innovative and participatory approaches in managing their own health.

According to the presented results, the developed product reinforces the potential of the intervention as part of perioperative care protocols. Previous studies have shown that the intake of liquids containing simple carbohydrates not only reduces discomforts such as hunger and thirst, but also attenuates metabolic responses to trauma, decreases insulin resistance, and may promote better clinical outcomes.^3^^,^^10^^,^^27^ Zhang et al.^28^ observed that preoperative carbohydrate use was associated with a near-significant reduction in the overall rate of perioperative complications in patients undergoing abdominal surgeries. Furthermore, the administration of an oral carbohydrate load demonstrated benefits for patient well-being, such as relief of thirst and reduced anxiety, as well as a marked decrease in postoperative metabolic and inflammatory responses, promoting a faster recovery after the surgical procedure.

Based on the results of Wang et al.^29^, the oral administration of low-concentration carbohydrates before surgery may promote a better quality of postoperative recovery, according to patient self-assessment, as well as reduce the incidence of postoperative hyperglycemia following thyroidectomy.

This study has some limitations that should be acknowledged. First, the control group did not receive a placebo clear beverage. Although standard fasting care was maintained, the absence of a placebo drink may limit the ability to fully distinguish the specific effects of carbohydrate administration from potential expectancy or hydration effects. Future randomized trials incorporating a placebo-controlled design would strengthen internal validity.

Second, the groups were not fully comparable regarding gender distribution, with a higher proportion of female participants in the test group than in the control group. Although gender is not expected to substantially influence satiety perception under these conditions, this imbalance may represent a potential confounding factor and should be considered when interpreting the findings.

Third, the sample size was relatively small and derived from a convenience sample. Although adequate for detecting differences in the primary outcome (satiety score), the study was not designed or powered to evaluate safety outcomes. This study was not powered to evaluate rare adverse events such as pulmonary aspiration. No adverse events were observed during the study period; however, this finding should be interpreted descriptively and should not be considered definitive evidence of safety. Larger studies specifically designed to assess safety outcomes are needed to further evaluate the safety profile of preoperative carbohydrate beverages.

## Conclusion

The instant coffee-flavored carbohydrate beverage was well accepted and was associated with favorable patient-reported outcomes when administered as a clear liquid during the preoperative period. Its intake was associated with reduced perception of hunger, thirst, and anxiety when compared with standard fasting care, contributing to greater patient-reported comfort. The product also demonstrated practical ease of preparation and a composition consistent with current nutritional recommendations. No adverse events were observed during the study period; however, the study was not powered to evaluate rare adverse events, and these findings should not be interpreted as definitive evidence of safety.

Although carbohydrate-containing beverages in the preoperative period are supported by scientific evidence, their implementation remains limited in many Brazilian hospitals. The present findings suggest a potential role for accessible and practical strategies aimed at reducing prolonged fasting and improving the perioperative experience. Broader adoption of evidence-based perioperative practices may help enhance patient-centered care, although larger studies are needed to further evaluate clinical outcomes and safety.

## Ethics approval and consent to participate

The clinical phase of this study was registered in the Brazilian Registry of Clinical Trials (ReBEC; RBR-5qczxhb – https://ensaiosclinicos.gov.br/rg/RBR-5qczxhb) and approved by the Human Research Ethics Committee (CAAE 67767223.9.0000.5351). The study was conducted in accordance with the Declaration of Helsinki. All participants provided written informed consent prior to enrollment.

## Consent for publication

Not applicable.

## Data availability statement

The datasets generated and/or analyzed during the current study are available from the corresponding author upon reasonable request.

## Declaration of generative AI and AI-assisted technologies in the writing process

During the preparation of this work, the authors used ChatGPT (OpenAI) to assist with language editing, grammar correction, readability improvement, and organization of the manuscript. All AI-assisted outputs were critically reviewed, edited, and validated by the authors, who take full responsibility for the accuracy, integrity, and scientific content of the published article.

## Authors’ contributions

Joice Carla Dorneles Zanin: Methodology; analyses; investigation; validation; writing-original draft. Rosicler Colet: Methodology; analyses; investigation. Marcieli Peruzzolo: Methodology; investigation; validation. Geciane Toniazzo Backes: Conceptualization; investigation; data analysis; writing-review & editing. Jamile Zeni: Conceptualization; investigation; data analysis; writing-review & editing. André Keng Wei Hsu: Conceptualization; methodology; investigation; data analysis; visualization; writing-review & editing; project supervision.

## Funding

No specific funding was received for the conduct of this study. However, support for open access publication was provided by the Coordination for the Improvement of Higher Education Personnel (CAPES-ROR: 00 × 0ma614), Brazil.

## Conflicts of interest

One or more authors are listed as inventors on the patent deposit BR102022022016-6 related to the beverage formulation evaluated in this study. No financial compensation or commercial licensing agreements are currently in place. All other authors declare no competing interests.
